# Coexpression of SFRP1 and WIF1 as a Prognostic Predictor of Favorable Outcomes in Patients with Colorectal Carcinoma

**DOI:** 10.1155/2014/256723

**Published:** 2014-05-14

**Authors:** Shiyong Huang, XiaoMing Zhong, Jun Gao, Rongfeng Song, Hongyu Wu, Shuming Zi, Shijie Yang, Peng Du, Long Cui, Chun Yang, Zikang Li

**Affiliations:** ^1^Department of Anorectal Surgery and Colorectal Cancer Center, Xinhua Hospital, School of Medicine, Shanghai Jiao Tong University, No. 1665 Kongjiang Road, Shanghai 200092, China; ^2^Department of Tumor Radiotherapy and Chemotherapy, Jiangxi Tumor Hospital, Nanchang, China; ^3^Department of Gastroenterology, Changhai Hospital, Second Military Medical University, Shanghai, China; ^4^Department of Gastrointestinal Surgery, Sichuan Academy of Medical Sciences and Sichuan Provincial People's Hospital, Chengdu, Sichuan, China; ^5^Department of Oncology, Ya'an People's Hospital, No. 358 Chenghou Road, Ya'an, Sichuan 610072, China

## Abstract

Colorectal tumorigenesis is ascribed to the activity of Wnt signaling pathway in a ligand-independent manner mainly through APC and CTNNB1 gene mutations and in a ligand-dependent manner through low expression of Wnt inhibitors such as WNT inhibitory factor 1 (WIF1) and secreted frizzled related protein 1 (SFRP1). In this study we found that WIF1 protein expression was increased and SFRP1 was decreased significantly in CRC tissue versus normal tissue, and high expression of WIF1 was associated with big tumor diameters and deep invasion, and loss of SFRP1 expression was associated with the left lesion site, deep invasion, and high TNM stage. Among the four expression patterns (WIF+/SFRP1+, WIF+/SFRP1−, WIF−/SFRP1+, and WIF−/SFRP1−) only coexpression of WIF1 and SFRP1 (WIF+/SFRP1+) was associated with favorable overall survival, together with low TNM stage, as an independent prognostic factor as shown in a multivariate survival model. The results indicated that WIF1 seemed to play an oncogenic role, while SFRP1 seemed to play an oncosuppressive role although both of them are secreted Wnt antagonists. Coexpression of SFRP1 and WIF1, rather than SFRP1 or WIF1 alone, could be used, together with low TNM stage, as a prognostic predictor of favorable outcomes in CRC.

## 1. Main Text

Colorectal cancer (CRC) is a common malignant tumor worldwide, and the incidence of which has increased rapidly over the past decade. Although various environmental risk factors have been found to play some role in tumorigenesis and the progressive accumulation of genetic and epigenetic alterations has proved to affect the key cellular signaling pathways that drive the transformation and progression of normal colonic epithelial cells to cancer cells, the pathological mechanism at the molecular level remains elusive [1, 2].

Wnt pathway is a critical regulator in embryonic development and maintenance of gut homeostasis. The transduction of Wnt signals between cells proceeds via a complex series of events, including posttranslational modification and secretion of Wnts, binding to transmembrane receptors, activation of cytoplasmic effectors, and transcriptional regulation of target genes [3, 4]. Aberrant regulation of the Wnt signalling pathway has therefore been suggested to play a role in tumorigenesis [5, 6], especially in the pathogenesis of CRC [7]. The Wnt pathway can be activated aberrantly not only by mutations in APC but also by CTNNB1 gene encoding b-catenin, leading to ligand-independent Wnt signaling [8, 9]. However, increasing evidence suggests that dysregulation of Wnt signaling by secreted antagonists on the cell surface is also associated with tumorigenesis [10–15]; for example, the low expression induced by promoter methylation of Wnt antagonists genes SFRP1 and WIF1 may induce ligand-dependent Wnt signaling activity [16–19].

Although the aberrant expression of SFRP1 and WIF1 as Wnt inhibitors for both canonical and noncanonical pathways was reported to be involved in the tumorigenesis of CRC, their protein expression patterns, their mutual association, and their correlations with various pathological and molecular features and the prognosis remain unclear. The present study provided the first evidence that SFRP1 and WIF1 were differentially expressed in CRC, and their coexpression, rather than SFRP1 or WIF1 alone, was associated with a favorable prognosis.

## 2. Material and Methods

### 2.1. Tissue Samples

CRC tissue specimens (*n* = 145) and adjacent normal mucosal specimens (*n* = 20) were obtained from surgical resection in the Department of Pathology of Xinhua Hospital affiliated to Shanghai Jiao Tong University School of Medicine (Shanghai, China) between March 2009 and June 2010. The demographic data of the subjects are shown in Table 1. No patient received chemotherapy or radiotherapy prior to specimen collection. All the pathological sections were reviewed by two pathologists (LC and HL) independently, and the final diagnosis was confirmed by pathology. Clinical characteristics included gross pathology, tumor location, tumor diameter, architectural features of the tumor tissue, WHO classification, grade, invasion, lymph node metastasis, liver metastasis, and stage. The study protocol was approved by the Ethics Committee of the said hospital.

### 2.2. Immunohistochemistry (IHC) Analysis and Evaluation

Tissue specimens were embedded in paraffin, sliced into 4 *μ*m sections, deparaffinized with xylene, hydrated in serial dilutions of alcohol, and immersed in 3% H_2_O_2_ to inhibit endogenous peroxidase activity. Then, the sections were incubated with the primary antibodies (anti-SFRP1 antibody, 1 : 800; Abcam, Cambridge, MA, and anti-WIF1 antibody, 1 : 50; Abcam, Cambridge, MA) overnight at 4°C, washed with phosphate buffer saline (PBS), and incubated with secondary antibodies and 3,3′-diaminobenzidine (DAB) color reagent (Supervision anti-rabbit detection reagent, Shanghai Long Island Biotec. Co., Shanghai, China). The sections were counterstained with Mayer's hematoxylin, dehydrated, and mounted with Canada balsam. Human lung carcinoma tissue and human breast cancer tissue from the Department of Pathology of Xinhua Hospital were used as positive control for primary antibodies WIF1 and SFRP1, respectively, and those treated with PBS were regarded as negative control.

Immunohistochemical SFRP1 and WIF1 expression was independently analyzed by two pathologists (SZ, JG) without the knowledge of the histopathological data. Cytoplasmic staining of WIF1 was confirmed as positive according to the previous reports [20–22] and the recommendation of the antibody production company (Abcam, ab71205) and that of SFRP1 was confirmed according to the previous reports [23, 24] and the recommendation of the antibody production company (Abcam, ab4193). The intensity of cytoplasmic staining was scored from 0 to 4+ (0: negative; 1+: weak; 2+: mild; 3+: moderate; and 4+: intense staining). The extent of staining was scored from 0 to 4+ (0: negative; 1+: 0–25%; 2+: 26–50%; 3+: 51–75%; and 4+: 76–100%) according to the percentage of the positively stained area. The product of the intensity and the extent of staining yielded final scores ranging between 0 and 16. Tumors with a final immunoreactivity score (IRS) ≤ 2 were considered as negative (0); 2 < IRS ≤ 4 as weakly positive (1+); 4 < IRS ≤ 9 as moderately positive (2+); and 9 < IRS as strongly positive (3+).

### 2.3. Statistical Analysis of Clinicopathologic Patient Data

Statistical analyses were completed using SPSS version 15.0 (SPSS, Chicago, IL, USA). Differences were considered statistically significant when *P* < 0.05. The difference of the mean variable between the groups was tested by one-way ANOVA test and the counting variable was tested by Chi-square test. The correlation between WIF1 and SFRP1 expression in CRC tissue was tested by Pearson test. Associations with overall survival (OS) were analyzed initially by Kaplan-Meier plots (log-rank test), and then Cox multivariate proportional hazards regression models were used to assess the OS power of these significant parameters.

## 3. Results

### 3.1. High Expression of WIF1 and Loss of SFRP1 Protein Expression in Human CRC Tissue

**Figure 1:**
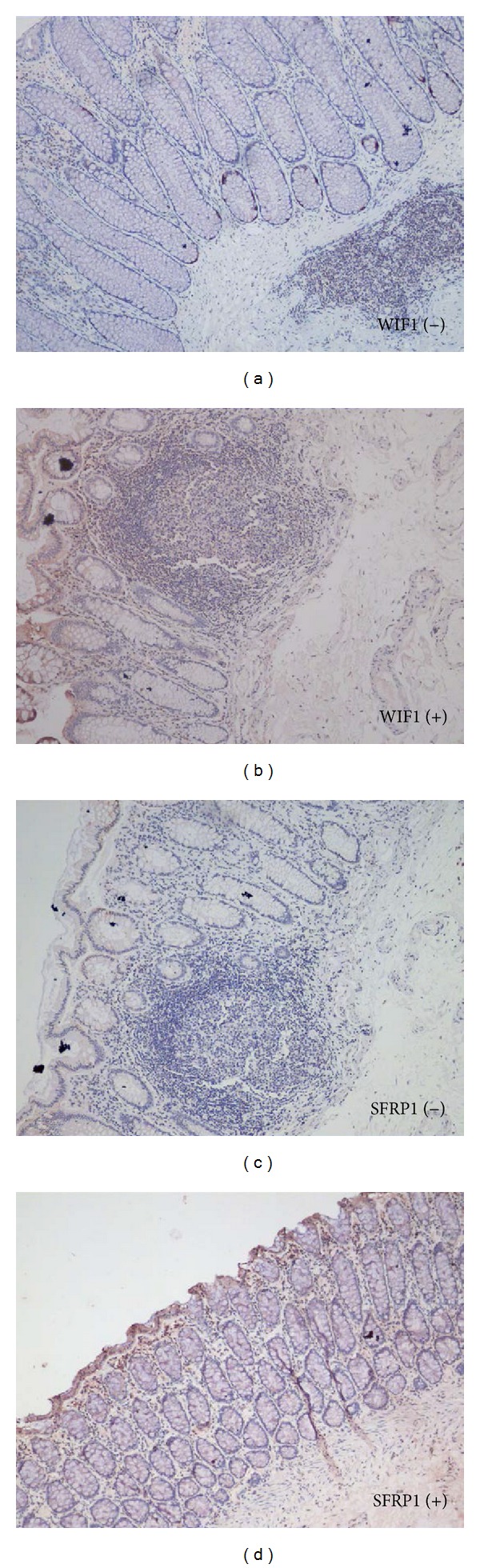
Representative photos of the positive and negative cytoplasmic staining of WIF1 and SFRP1 in normal colorectal mucosa. The immunohistochemical expression of WIF1 and SFRP1 is mainly present in the cytoplasm of epithelial cells of colonic mucosa in normal tissue (×100).

**Figure 2:**
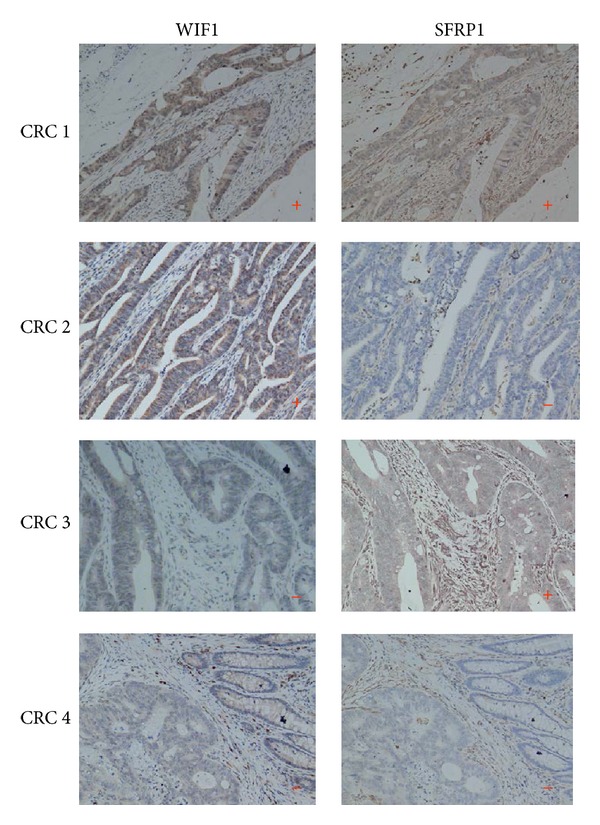
Immunohistochemical expression of WIF1 and SFRP1 in CRC tissue. The representative photos of the four expression patterns of WIF+/SFRP1+, WIF+/SFRP1−, WIF−/SFRP1+, and WIF−SFRP1− in CRC tissue (×100).

As shown in Figure 1, the positive staining of WIF1 and SFRP1 proteins was mainly present in the cytoplasm of epithelial cells of colonic mucosa in normal tissue. The positive rate of SFRP1 was 62.8% (91/145) in CRC tissue and 95% (19/20) in normal tissue, while the positive rate of WIF1 was 72.4% (105/145) in CRC tissue and 45% (9/20) in normal tissue. The semiquantitative evaluation data are shown in Table 2. The result of statistical analysis indicated that SFRP1 protein expression was decreased significantly and WIF1 was increased significantly in CRC tissue, showing a weak negative correlation between them (Table 3). The four expression patterns (WIF+/SFRP1+, WIF+/SFRP1−, WIF−/SFRP1+, and WIF−SFRP1−) in CRC tissue are shown in Figure 2.

### 3.2. Correlations between WIF1/SFRP1 Protein Expression and Clinicopathological Characteristics

The association of WIF1 and SFRP1 protein expression with clinicopathological features of CRC patients was determined. As shown in Table 4, high expression of WIF1 was significantly associated with big tumor diameters and deep invasion, while loss of SFRP1 expression was significantly associated with the left lesion site, deep invasion, and high TNM stage. No other significant association was observed otherwise.

### 3.3. Association of WIF1/SFRP1 Protein Expression with OS in CRC Patients

**Figure 3:**
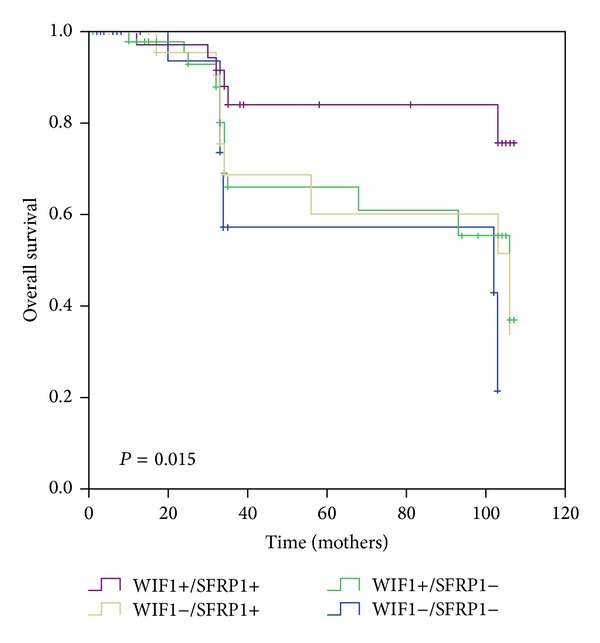
Kaplan-Meier plot: coexpression of SFRP1 and WIF1 is a predictor of overall survival in CRC patients and is significantly correlated with high expression of both SFRP1 and WIF1 and overall survival.

The 145 CRC patients were followed up for a median period of 34 (1–107) months, of whom 11 patients were lost to follow-up. Univariate survival analysis was used to assess the impact of clinicopathological characteristics and WIF1 and SFRP1 protein expression on patient survival. As shown in Figure 3 and Table 5, coexpression of SFRP1 and WIF1 was significantly associated with favorable OS (the median OS was 95 months for WIF1(+)/SFRP1(+)*  *versus*  *77.1 months for the other patterns). Some pathological features including invasion, lymph metastasis, liver metastasis, and high TNM stage were significantly associated with poor OS. Multivariate survival analysis by inputting significant variants from the univariate survival analysis into the Cox regression model showed that coexpression of SFRP1 and WIF1, together with low TNM stage, was an independent prognostic factor for favorable OS (Table 5).

## 4. Discussion 

It was observed in the current study that WIF1 and SFRP1 proteins had aberrant expression patterns in CRC tissue, with upregulation of WIF1 and downregulation of SFRP1, and that there was a weak negative correlation between them. In addition, high expression of WIF1 was significantly associated with big tumor diameters and deep invasion, while loss of SFRP1 expression was significantly associated with the left lesion site, deep invasion, and high TNM stage, implying that WIF1 and SFRP1 play different roles in the tumorigenesis of CRC, though both of them belong to similar secreted inhibitors of the Wnt pathway.

In a normal physiological state, Wnt proteins (a large family of palmitoylated secreted glycoproteins as ligands) activate the Wnt pathway via at least three different pathways by binding to the ligand-receptor: the canonical pathway through Wnt, Fz, LRP5/6, and *β*-catenin; the noncanonical pathway through Wnt, Fz, small GTPase RhoA, and Rac for planar cell polarity; and through Wnt and Fz mediated release of Ca^+2^ from intracellular stores [25]. There are many different levels of regulation in the Wnt signaling pathway at many different places: outside cells, at the outer surface of the cell membrane, at the inner surface of the cell membrane, in the cytoplasm, and in the nucleus [25]. The primary regulatory phase for Wnt activity is at the cell surface by different transmembrane proteins, and the secretion of Wnt proteins from cells is promoted by Wntless [26]. Once Wnt proteins are secreted from cells, the modification by glycosaminoglycans and lipid modulates their distribution, diffusion, and signal transduction [27]. At the same time, the activation of Wnt signaling is further controlled by the antagonists of two functional families: one is the secreted frizzled-related protein (SFRP) family, including SFRP family, WIF1, and cerberus that can directly bind to Wnt proteins to inhibit the canonical and noncanonical pathways, and the other is the dickkopf family, including the dickkopf proteins that can directly bind to the LRP5/LRP6 component of the Wnt receptor complex, thus specifically inhibiting the canonical pathway [28].

The aberrant activity of the Wnt pathway occurs in several malignancies via multiple genetic mechanisms [29]. Both mutations in beta-catenin interfere with its phosphorylation and degradation, and the loss of functional mutations in APC destabilizes the Axin-APC complex, causing accumulation of beta-catenin protein in the cell nucleus. Such APC mutations occur in about 80% of human CRC, resulting in ligand-independent activation of canonical Wnt signaling associated with the loss of controlled growth and the impairment of cell differentiation. Indeed, these mutations occur in a large proportion of tumors and are thought to cause the downstream signaling independent of upstream signals [30].

However, the present study addressed a more important issue concerning the Wnt pathway underlying the pathogenic mechanism: whether what happens at the cell surface would influence CRC tumorigenesis. Recently, involvement of upstream signal regulation in CRC has been reported, suggesting that the Wnt signaling pathway may be regulated in a quantitative manner at different levels [31]. For example, the loss of SFRP family expression was associated with promoter hypermethylation in CRC [19, 32]. In addition, the restoration of SFRPs in colon cancer cell lines carrying CTNNB1 or APC mutations resulted in the suppression of Wnt-dependent transcription and a higher rate of apoptosis, while the overexpression of Wnt-1 in CTNNB1 mutant cell lines increased Wnt-dependent transcription [19], and blocking Wnt-1 signaling induced apoptosis in human CRC cells containing downstream mutations [31]. These reports are in agreement with our data, supporting the idea that activation of the Wnt pathway receptor at the cell surface would enhance propagation of the signal caused by alterations in the mutated components, which would further induce the crosstalk between the tumorigenic canonical and noncanonical JNK signalling pathways [25].

SFRP1 and WIF1 belong to the same class and have a similar function of binding to Wnt proteins to inhibit the canonical Wnt signaling. However, few previous studies have reported the dissimilarity of SFRP1 and WIF1 in CRC tumorigenesis. For the first time, our data demonstrated that the expression patterns of SFRP1 and WIF1 proteins were different in CRC tissue. The loss of SFRP1 expression in CRC tissue as we found in the present study is consistent with the previously proposed opinion that constitutive Wnt signaling may be required to complement downstream mutations in the evolution of CRC [23, 25]. More importantly, the present study first reported the upregulation of WIF1 expression in CRC tissue.

The finding that WIF1 expression was upregulated in CRC tumorigenesis urged us to speculate the molecular pathogenic mechanism. Unlike SFRPs, there is little evidence to support the role of WIF-1 as a tumor suppressor at present. Although some reports showed that both mRNA and protein expressions of WIF1 were downregulated in prostate, breast, lung, and bladder cancers, hypermethylation of CpG islands in the WIF1 promoter region has been observed in some types of cancer [3, 18, 20], suggesting that loss of WIF1 expression may also contribute to carcinogenesis. On the other hand, several other reports offered a different view about the expression and activity of secreted Wnt antagonists in the tumor setting [33], saying that the upregulation of WIF-1 expression was detected in colonic adenoma and CRC cell lines, along with the loss of expression of other inhibitors such as SFRPs and Dkk-1 [34], and that the Drosophila ortholog of WIF-1 facilitated hedgehog diffusion, thus raising the possibility that mammalian WIF-1 may enhance hedgehog activity [35, 36]. Nevertheless, based on this study, we conjectured that upregulation of WIF-1 expression in CRC tissue may depend on other processes independent of Wnt signaling, which seems to be a logical explanation and warrants further study.

Colorectal carcinoma is the second leading cause of cancer death worldwide, causing an average of 50,000 deaths per year. The exploration of the nonanatomic prognostic factors into the current TNM staging system is urgently needed and could improve the understanding of colon cancer on the cellular basis and the role of signaling pathways in CRC tumorigenesis [37, 38]. In this study, we observed that the two similar Wnt secret inhibitors WIF1 and SFRP1 had significantly different expression patterns in CRC tissue and that these different expression patterns were correlated with various clinical pathological subtypes. WIF1 seems to have an oncogenic effect, while SFRP1 seems to have a suppressive effect. More importantly, coexpression of SFRP1 and WIF1, rather than SFRP1 or WIF1 alone, is a prognostic factor predicting a favorable outcome in CRC. These findings suggest that the secreted WNT antagonists may be useful molecular markers for CRC classification and assessment of the therapeutic target. The regulatory mechanisms of the different expression patterns of SFRP1 and WIF1 need to be further investigated.

## Figures and Tables

**Table 1:** Patient demographic data.

Characteristics	Normal (*n* = 20)	Cancer (*n* = 145)	*P* value
Age (mean ± SD, year)	67 ± 10	66 ± 14	0.907^a^
Gender			
Male	9	65	0.998^b^
Female	11	80	
Lesion sites			
Left	10	58	0.394^b^
Right	10	87	

^a^One-way ANOVA test; ^b^Chi-square test.

**Table 2:** Semiquantitative evaluation of WIF1 and SFRP1 protein expression in normal and CRC tissues.

Group	Cases	WIF1 expression staining score				SFRP1 expression staining score			
		0	1	2	3	0	1	2	3
Normal	20	11	7	2	0	1	7	5	7
	% in group	55.0	45.0			5.0	95.0		

CRC	145	36	59	32	18	54	74	10	7
	% in group	24.8	75.2			37.2	62.8		

Normal versus CRC		Chi-square test, *P* = 0.000				Chi-square test, *P* = 0.000			

**Table 3:** Correlations between WIF1 and SFRP1 expression in 145 CRC tissue specimens.

	SFRP1		Pearson's *R*	*P* value
	+	−		
WIF1				
+	40 (38.1%)	65	−0.197	0.017
−	24 (60.0%)	16		

**Table 4:** Correlations between WIF1/SFRP1 protein expression and clinical characteristics of CRC patients.

Characteristics	Cases (*N* = 127)	Immunoreactive positive (%)			
		WIF1	*P* value	SFRP1	*P* value
Age (year)			0.652		0.704
≤65	66	49 (74.2)		28 (42.4)	
>65	79	56 (70.9)		36 (45.6)	
Gender			0.273		0.115
Male	80	55 (68.8)		40 (50.0)	
Female	65	50 (76.9)		24 (36.9)	
Lesion sites			0.704		**0.000**
Right	87	62 (71.3)		49 (56.3)	
Left	58	43 (74.1)		15 (25.9)	
Architectural features			0.488		0.835
Mucus	15	12 (80.0)		7 (46.7)	
Nonmucinous	130	93 (71.5)		57 (43.8)	
Tumor diameter			**0.005**		0.250
≤4.5 cm	67	41 (61.2)		33 (49.3)	
>4.5 cm	78	64 (82.1)		31 (39.7)	
Invasion			**0.001**		**0.025**
T1	6	2 (33.3)		5 (83.3)	
T2	19	15 (78.9)		9 (47.4)	
T3	41	22 (53.7)		23 (56.1)	
T4	79	66 (83.5)		27 (34.2)	
Lymph metastasis			0.096		0.132
N0	84	61 (72.6)		43 (51.2)	
N1	24	21 (87.5)		8 (33.3)	
N2	37	23 (62.2)		13 (35.1)	
Liver metastasis			0.703		0.109
Negative	132	95 (72.0)		61 (46.2)	
Positive	13	10 (76.9)		3 (23.1)	
TNM stage^a^			0.878		**0.042**
I	29	21 (72.4)		16 (55.2)	
II	52	39 (75.0)		27 (51.9)	
III	47	34 (72.3)		18 (38.3)	
IV	17	11 (64.7)		3 (17.6)	

^a^According to the 2002 version of the American Joint Committee on Cancer (AJCC) and the International Union Against Cancer (UICC).

**Table 5:** Kaplan-Meier and Cox multivariate proportional hazard analysis for clinicopathological characteristics of CRC patients.

Characteristics	Univariate analysis log-rank	*P*	Multivariate analysis hazard ratio (95%)	*P*
Age (year)				
≤65	1.047	0.306		
>65				
Gender				
Male	0.407	0.523		
Female				
Lesion sites				
Right	2.627	0.105		
Left				
Architectural features				
Mucus	3.227	0.072		
Nonmucinous				
Tumor diameter				
≤4.5 cm	0.032	0.858		
>4.5 cm				
Invasion				
T1-T2	5.579	0.018		NS
T3-T4				
Lymph metastasis				
N0	9.773	0.002		NS
N1-N2				
Liver metastasis				
Negative	5.583	0.018		NS
Positive				
TNM stage^a^				
I-II	10.983	0.001	0.391 (0.201–0.760)	0.006
III-IV			1	
WIF1 and SFRP1 expression				
WIF(+)/SFRP1(+)	5.477	0.019	0.428 (0.187–0.983)	0.045
The others			1	

^a^According to the 2002 version of the American Joint Committee on Cancer (AJCC) and the International Union Against Cancer (UICC).

NS: variables not significant in the equation, *P* > 0.05.
